# Cognitive performance is linked to survival in free-living African striped mice

**DOI:** 10.1098/rspb.2023.0205

**Published:** 2023-03-08

**Authors:** Celine Rochais, Carsten Schradin, Neville Pillay

**Affiliations:** ^1^ School of Animal, Plant and Environmental Sciences, University of the Witwatersrand, Braamfontein 2000, Johannesburg, South Africa; ^2^ IPHC, UNISTRA, CNRS, 23 Rue du Loess, 67200 Strasbourg, France

**Keywords:** cognition, evolution, fitness, survival, rodents

## Abstract

Cognition is shaped by evolution and is predicted to increase fitness. However, the link between cognition and fitness in free-living animals is unresolved. We studied the correlates of cognition and survival in a free-living rodent inhabiting an arid environment. We tested 143 striped mice (*Rhabdomys pumilio*) using a battery of cognitive tests, including: (i) an attention task, (ii) two problem-solving tasks, (iii) a learning and reversal learning task, and (iv) an inhibitory control task. We related cognitive performance with days of survival. Better problem-solving and inhibitory control performance were significant correlates of survival. Surviving males showed greater reversal learning which may be related to sex-specific behavioural and life-history characteristics. Specific cognitive traits and not a composite measure of general intelligence underpins fitness in this free-living rodent population, enhancing our understanding of the evolution of cognition in non-human animals.

## 1. Background

Why some individuals learn more quickly than others, memorize or perform better in several cognitive tasks are questions that have been intensely studied in humans, leading to the suggestion of the concept of ‘general intelligence’ [1,2]. This concept is hypothesized to underlie all cognitive abilities and is defined by a construct including reasoning ability and behavioural flexibility, underpinned by general learning, memory and executive functions [3]. Such general intelligence is a predictor of life outcomes, such as health and survival in humans [3]. The relationship between general intelligence and fitness measures raises the question of the evolutionary origin of general intelligence and its existence in non-human animals to understand how natural selection shapes the evolution of cognition.

The concept of human general intelligence is built on a positive correlation between performances across tasks of different cognitive domains, i.e. the positive manifold [4]. Factor-analytical procedures are used to reveal a single factor that loads positively for all cognitive tasks and that can explain a significant amount of variation (greater than 30% of variance), often termed ‘*g*’ or ‘*g factor*’ for general intelligence [5]. Although the biological significance of the human *g* factor is still debated [6], methods based on a cognitive battery of tests to create a *g* factor have been suggested as a powerful approach for identifying the existence of general intelligence in non-human animals (e.g. [7]). For example, a *g* factor has been identified in laboratory mice (*Mus musculus*) tested across a variety of tasks, such as maze navigation and sensory discrimination (e.g. [8–10]). Other studies suggested the existence of a *g* factor in wild species, using tasks testing different cognitive domains (e.g. Australian magpies (*Cracticus tibicen dorsalis*): [11]; bower birds (*Ptilonorhynchus violaceus; Ptilonorhynchus maculatus*):[12,13]; North Island robin (*Petroica longipes*), [14]). However, contrasting results showed that performance is not always positively correlated across domains in non-human animals. Other studies have reported negative correlations between acquisition and reversal learning (e.g. Indian myna, *Sturnus tristis*; [15]; Florida scrub-jays, *Aphelocoma coerulescens*; [16]), as well as between song complexity and inhibitory control and between song complexity and spatial learning (song sparrows; [17,18]). These contrasting results could highlight that cognition includes independent ‘modules’ that are domain-specific (e.g. [19]).

An array of cognitive traits impacts the behaviour of free-living animals, including perception, attention, learning, memory, decision-making and executive functions (flexibility, categorization, problem-solving [20]). Individual variation in cognitive performance is well documented in the animal kingdom [21,22], yet the evolutionary significance of cognitive variation remains poorly understood [23]. Cognitive performance can vary within and among species [24,25] because of the influence of environmental factors and is underpinned by selection for cognitive traits associated with fitness outcomes [26]. One approach to understand the link between cognition and fitness is to first measure cognitive performance in individuals and then correlate this with a fitness metric [27], such as reproductive success and/or survival [28,29]. For example, pioneering studies in birds showed a positive relationship between problem-solving performance and reproductive success, measured as clutch size or mating success [29,30].

Better cognitive performance is sometimes associated with fitness benefits [31,32] but not always [28,33]. For example, song repertoire size, an indirect predictor of various reproductive success metrics in birds, is positively correlated with inhibitory control (detour task) in male song sparrows (*Melospiza melodia*), but not with learning performance in a motor or colour discrimination task [17]. Furthermore, in some species, one domain-specific cognitive trait could be under strong selection compared to others, such as spatial memory in food-caching species [31] or flexibility in species living in an unpredictable environment [34]. To further our understanding of the evolution of cognition, it is of interest to explore several specific ecologically relevant cognitive traits underpinning behaviours directly linked to survival and reproduction in the wild [21].

Direct selection for cognitive traits is associated with greater innovation rates and brain size, which predict colonization and survival success in birds [35] and mammals [36]. Yet, most of these studies did not specifically test whether general intelligence in animals is directly related to survival success in the natural environment, and there is no evidence for a link between a *g* factor and reproductive success in wild birds [6,13]. Furthermore, it has been suggested that general intelligence results from a cluster of cognitive traits that are linked together and are elicited by evolutionary conditions and factors that create a set of selection pressures [37]. However, cognitive arrays are the result of species-typical adaptations to their whole ecological and social environments [3]. Thus, it raises the question of whether a *g* factor or specific cognitive traits instead better correlates with fitness in free-living animals with a specific ecological and social environment.

In the present study, we explored cognition–survival links in a free-living, diurnal rodent species, the African striped mouse, *Rhabdomys pumilio*. Striped mice are ideally suited for such a study because they have to cope with variable ecological and social environments [38]. Striped mice in our study population in the Succulent Karoo semi-desert areas in South Africa endure marked seasonal changes in food availability [39]. The frequency and intensity of droughts have increased in this area, and the Succulent Karoo biome has been identified as being particularly vulnerable to climate change [40]. The dry summer season is characterized by low food availability. Striped mice typically breed in the austral winter/spring and must survive the dry summer season to reproduce. Thus, striped mice have to respond flexibly to seasonal changes in food availability while encountering various challenges to survive, particularly predation risk [41]. Furthermore, striped mice show social flexibility (from group to solitary living) as an adaptation to unpredictably changing environments [38]. This shows the importance of social cognition skills, such as individual recognition [42], in this species which could be critical for survival.

Cognitive performance varies seasonally in striped mice: individuals tested in summer solved a new problem (a proxy of innovation [43]) faster compared to those tested in winter [44]. Furthermore, attention, spatial learning and memory, problem-solving and reversal learning varied seasonally by sex: in summer, males showed faster attention towards a predator stimulus, solved more novel problems, but made more errors and took longer in a spatial learning and memory tasks than males tested during winter [44,45]. By contrast, females tested in summer solved a reversal learning task faster but their attention, problem-solving and spatial learning and memory performance did not differ seasonally [44–46]. Female survival was correlated with faster attention to a predator stimulus, whereas male survival was correlated with greater spatial memory [47]. Therefore, cognition–survival links in striped mice could be related to sex-specific behavioural and life-history characteristics, such as male-biased dispersal.

We aimed to link the cognitive performance with survival, a proxy of fitness, in striped mice, using a novel battery of cognitive tests. The tests comprised five tasks: an attention task; two problem-solving tasks; a learning and reversal learning task; and an inhibitory control task. Our observations enabled us to assess whether general intelligence versus specific cognitive traits is adaptive in striped mice. We aimed to test multiple cognitive traits and investigate whether (i) a *g factor* can be detected, and (ii) relate both a *g factor* and specific cognitive traits to survival. We also considered the potential influence of covariates such as sex and body condition in order to understand the mechanisms involved in the evolution of cognition. We made two predictions:

— a *g factor* exists in striped mice as found in other species; and
— specific cognitive traits, such as problem-solving, instead of a general intelligence score, will be related to individual survival, since striped mice must respond flexibly to their environmental changes.

## 2. Methods

### (a) Study animals

We conducted our study in Goegap Nature Reserve, South Africa (29°41.56' S, 18°1.60' E) between 2018 and 2019. Our striped mice population was monitored continuously using behavioural observations, trapping and radiotracking [48]. Mice were trapped using baited metal live traps (26 × 9 × 9 cm) placed under bushes. Trapping sessions were usually conducted each morning (5 days a week) within the first hour after sunrise. Traps were checked 30 and 60 min after they were set. During trapping, mice were sexed and body mass (to ±1 g) and length (to ±1 mm, from tip of the nose to the anus) were measured. Mice were permanently marked with numbered metal ear tags (National Band and Tag Co., Newport, USA) and with commercial hair dye (Rapido, Pinetown, South Africa) for visual identification. Upon first capture after birth, body mass (if less than 30 g) and the date of first capture were used to estimate an individual's birth date using a species-specific growth curve [49]; from this date, the age of test individuals was determined. Age cannot be reliably estimated from weights over 30 g upon first capture, resulting in age data being unavailable for 18% of the tested mice (*n* = 27/143).

Cognition and survival were assessed in 143 (67 females, 76 males) adult mice. We related individual differences in cognitive performance tested with battery of tests, previously validated in other species (e.g. [7,11,50]), to individual mice survival [47]. We estimated the number of days that each test mouse survived from 1 January (beginning of the dry season) to 15 July (onset of the breeding season and before long-distance dispersal). A mouse was considered to have died when it had not been trapped or observed for at least two consecutive months. Thus, mice were classified as survivors (yes: if they were alive until 15 July) or else as non-survivors (no) [47].

### (b) Cognitive tests

**Figure 1.**
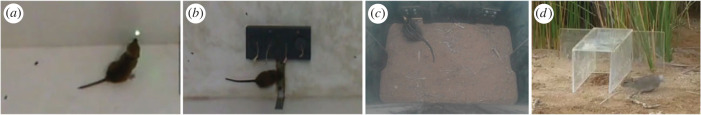
The test apparatuses, with a mouse for scale. Tests in the field laboratory: (*a*) visual stimulus used for the attention task; (*b*) the grid and lids used for the problem-solving task 1. Tests in the field at nest of mice: (*c*) escape box with two doors used for the problem-solving task 2 and the learning—reversal tasks; (*d*) the transparent trap-shape device with one end opened used in the inhibitory control detour task.

Cognitive tests were either performed in a field laboratory (e.g. attention and problem-solving 1) or directly in the field at the test individual's nest (e.g. problem-solving 2, learning and reversal, inhibitory control; figure 1). Each time a mouse was trapped, its identity and body mass were recorded. For laboratory tests, two–four striped mice were trapped in the morning (two rounds of trapping 30 min apart) at their nest within the first hour after sunrise. The laboratory (3.70 × 3.10 × 2.40 m) was located within a 5 min walking distance from the field site (see [44] for details). Each test was performed in the same unfamiliar arena made of white wood (80 × 65 × 94 cm). The arena was cleaned with 70% alcohol after each test. The time from capture in the trap to testing and final release to the nest was a maximum of 2 h.

#### (i) Attention task: visual attention test in the field laboratory

Following 5 min of habituation to the testing arena, mice were subjected to the visual attention task for 5 min (see also [51]). Their spontaneous visual attention was tested by using a standardized circular moving visual stimulus (Ø1 cm). A green laser light (Laser point, PEARL®) was projected on the opposite wall of the arena for 5 min (electronic supplementary material, S2; figure 1). The stimulus was chosen based on visual acuity of the mice (striped mice detect green light [52]) and the novel and non-frightening valence of the stimulus. The visual attention was later analysed from video footage. Rodents have a natural propensity to orient their gaze, the head or whole body, towards a salient stimulus in their environment [45]. A mouse was recorded to have shown an attentive response when it turned its head towards the visual stimulus. The total time spent being attentive to the stimulus was recorded frame by frame (frame, 0.02 s; electronic supplementary material, S1, table S1). We measured the total duration (in seconds) of gaze to the stimulus as a measure of attention, since it is representative of spontaneous attention towards environmental stimuli in the field and total duration was positively correlated to the number of attention sequences.

#### (ii) Problem-solving task 1: lid opening in the field laboratory

Immediately after the attention test, a lid opening problem-solving device was placed into the arena (electronic supplementary material S3). The device consisted of a grey PVC foraging grid (20 × 8 × 3.5 cm) containing four wells (each with a diameter of 3.5 cm and a depth of 2.5 cm) that were covered with grey PVC lids. A short cotton string (3 cm) was attached to each lid, which a mouse could pull to open the lid. Each well contained food reward consisting of a small piece of bran flake and a sunflower seed.

Mice were first trained for 5 min to search the wells, from where they could gain access to a food reward by first being exposed to the lids only partially covering the wells. After 5 min, we refilled the wells if they were empty, all the lids of the wells were closed again and the mouse was tested for 30 min. To provide the mice with sufficient time to perform the task in an unfamiliar environment, mice were tested on two consecutive days with the same lid opening procedure (i.e. 5 min habituation and 30 min of testing resulting in 1 h 10 min of total testing per individual over 2 days). Problem-solving performance was later analysed from video footage. We recorded the total number of lids opened (zero–four lids) as a measure of problem-solving performance (electronic supplementary material, S1, table S1).

#### (iii) Problem-solving task 2: escape box test in the field

Between 3 and 10 days after the lid opening problem-solving task, the mice were subjected to an escape box (door opening) task at their nests in the field. We used 135 of the 143 mice in the door opening task since eight mice were not re-trapped. After being trapped directly at their nests in the morning, mice were placed in a black testing box (40 × 30 × 35 cm) equipped with a transparent perpex lid to prevent mice from jumping out of the box. The box had two black doors that opened freely, situated 17 cm apart on the same side of the box (electronic supplementary material, S4). The door system was designed to allow a mouse to escape and return to its nest (i.e. the reward stimulus) by pushing the door with its head and/or legs. The escape box was located 50 cm in front of the nest, with the doors facing the nest. The subject was videotaped using a GoPro camera (AEE LYFE, S91B) mounted on the top of the box. Subjects were given a maximum of 5 min to open the door, whereafter those that did not complete the task were released to their nests. Mice were tested repeatedly at each trapping session until they opened either door on three successive attempts, which all mice accomplished in three–six trials over several days depending on how often they were trapped (mean ± s.e.m. = 3.17 ± 0.08 trials). A trial was defined as each time a mouse was trapped and placed into the apparatus. A mouse was tested only once per trapping session because the incentive was to return to its nest. We measured the number of trials taken to open the door until reaching three successive successes (from three to six) as a measure of problem-solving performance (electronic supplementary material, S1, table S1).

#### (iv) Learning and reversal learning tasks: escape box test in the field

One day after the completion of the problem-solving task 2, mice were tested directly at their nest at each trapping session in the field in a sequential learning–reversal learning task in which they had to choose between opening a left or right door of the escape box to reach their nests (electronic supplementary material, S5). After being trapped directly at their nests (during the normal trapping sessions), mice were placed in the same black testing box as in the problem-solving task 2 (see above). Mice were given a maximum of 5 min to complete the task whereafter those that did not complete the task were released [53]. Each mouse was first tested three times with both doors open and could swing freely (i.e. initial spatial preference with the problem-solving task 2) and the door (right or left) it selected 2 out of 3 times was assigned as its preferred one [54]. Once a mouse ended the initial spatial preference test (100% of tested mice), it was subjected to the learning task at the next trapping session, where the preferred side was kept open (‘door open’ to reach the nest) and the non-preferred side was locked shut from the outside (‘door closed’ from the outside to prevent visual cues of the lock). An individual was considered to have succeeded at the learning task when it reached the learning criterion of selecting its preferred door at least 10 out of 12 consecutive trials. A mouse was provided with as many trials as necessary to reach the learning criterion (range: 12–23 trials). A trial was considered to be each time a mouse was trapped and placed into the apparatus. A mouse was tested only once per trapping session because the incentive was to return to its nest.

During the next trapping session after the completion of the learning task (e.g. the following day), the reversal learning test began when the incentive (door) was switched spatially (electronic supplementary material, S6). The initially preferred side was locked shut (door closed) and the previous non-preferred side was rewarded (door opened). Mice had to reach the same learning criterion of at least 10 out of 12 consecutive correct choice trials. Again, a mouse was provided with as many trials as possible until it reached the 10 correct consecutive trials criterion (range: 12–24 trials).

For each experimental task (learning task and reversal learning task), we assessed the accuracy of mice in each trial, by measuring whether the response was correct or incorrect. Next, we computed the accuracy (in per cent) for all trials as the number of correct responses by mice divided by the total number of responses made as a measure of performance efficiency (electronic supplementary material, S1, table S1).

#### (v) Inhibitory control task: detour test in the field

Mice were tested in a detour task at their nest in the field to quantify their ability to inhibit ineffective proponent responses towards food. Mice from the same group were tested in the evening, for 30 min over 4 days to increase the probability of observing the spontaneous behaviour at the nest and to observe a mouse interacting with the device alone (to limit social learning influence and/or food competition during the task). Each individual was visually identified from their colour markings on the pelage. The apparatus consisted of a rectangle with an open end on one side and a transparent open-ended wall on the other side; the device was similar to that of the traps to which the mice were accustomed (26 × 9 × 9 cm). The apparatus was placed 50 cm in front of the nest and a food reward (i.e. five pieces of the normal bait) was placed in the centre of the apparatus (electronic supplementary material, S7). The device walls had holes to allow the mice to smell the food incentive. As far as possible, the test subjects were presented with the task such that the open ends of the device faced away from the nest: mice usually came out of the nest, facing the closed (transparent) part of the device and had to use a detour to gain access to the opened part of the device to reach the food reward. Mice were videotaped using a casio HD web camera mounted on a tripod, located 5 m away from the focal nest. A trial (i.e. an attempt to reach the food reward) was deemed successful if the test subject inhibited the intended movement towards the closed, transparent part (i.e. end and sides) of the device and detoured around to the open ends of the device to gain access to the food reward. An unsuccessful attempt was considered when the mouse did not inhibit its movement towards the closed, transparent part. We recorded the number of attempts (i.e. the number of unsuccessful attempts until success) taken to succeed in the task as measures of inhibitory control performance (electronic supplementary material, S1, table S1). Other studies using the detour task commonly include a training/familiarization phase in which test subjects are presented with an opaque apparatus before being exposed to the transparent one (for example, [17]). We considered that mice were accustomed to the opaque apparatus since they were experienced with the opaque traps during the usual trapping.

### (c) Data analyses

The video recordings of the tests were scored using the Kinovea (Kinovea 0.8.15) software. Scoring was done by the same observer (C.R.) and also by one field assistant who was naive to the experimental treatment (10% of test sessions, inter-observer reliability—Spearman correlation rank test, *N*_mice_ = 12, rs = 0.85). All statistics were performed with R v. 3.6.1 [55]. The significance level was set at 0.05. Descriptive statistics are reported as means, standard deviation (s.d.) and coefficient of variation (CV). Independence and homogeneity of variances of the models were assessed by inspection of fitted residuals using the *plotresid* function in the *RVAideMemoire* package [56]. Linear models were constructed using the lm/glm function in the *lme4* package [57], and statistical tests were performed using the Anova function in the *car* package [58]. Statistical tests (likelihood ratio test) were performed with loglink function for Gaussian and Poisson distribution and logitlink function for binomial distribution. *Post hoc* tests were performed using the *emmeans* function in the *emmeans* package [59], along with the contrast pairwise comparison function (emmeans, ‘pairwise’) using a *t*-test with a Tukey correction. Principal component analysis (PCA) was performed using the *FactomineR* package [60].

We analysed individual differences in cognitive performance for each cognitive task separately. We assessed the influence of intrinsic characteristics using linear models (LM) and generalized linear models (GLM) with cognitive performance as the dependent variable and sex, body mass as fixed effect and age as a covariate.

We assessed how the performance in one task related to the performance in another task. First, we assessed simple relationships between performance in pairs of tasks using an ordinary least rectangles regression for each variable as interdependents and testing for factors which could predict the relationship [56]: regression < least rect (problem-solving 1 ∼ problem-solving 2 | sex).

Second, we assessed whether inter-individual variation in performance across cognitive tasks could be explained by a composite underlying factor (analogous to *g*), by conducting a PCA on scores from the 143 mice tested with cognitive tasks. We conducted a PCA with an unrotated factor solution and extracted individual composite scores from the first, second and third components with eigenvalues greater than 1. The scores from the first unrotated principal component are widely used as a measure of *g* in both humans and non-human animals [13,61]. Previous studies have inferred the existence of *g* when all task performances load positively onto the first unrotated component extracted that also explains 30–45% of the variance in cognitive performance [13].

We assessed the relationship between cognitive performance in each cognitive task and survival using a survival regression which accounted for the censored value of survivor (censoring status 0 = censored, 1 = dead observed) and the increase of instantaneous risk of death over time [56]. We created an object to model individual mice survival as:

$$\begin{array}{rl} \mathrm{Survival}<- & \mathrm{Surv}(\mathrm{number}\;\mathrm{of}\;\mathrm{days}\;\mathrm{of}\;\mathrm{survival}\;\mathrm{before}\;\\ & \mathrm{dispersal},\;\mathrm{censoring}\;\mathrm{status}) \end{array}.$$

The survival regression aimed to explain which factors predict the created survival object and is based on Weibull distribution used to model the evolution of instantaneous risk of death over time [62]. We assessed the influence of cognitive performance for each cognitive task, the interaction between cognition and sex (since we found sex differences in cognition) as an explanatory variable, and the influence of body mass and age as covariates:

$$\begin{array}{rl} \mathrm{model}\;1<- & \mathrm{survreg}(\mathrm{survival}∼\mathrm{cognitive}\;\mathrm{performance}\times \mathrm{sex}\\ & +\mathrm{body}\;\mathrm{mass}+\mathrm{age},\mathrm{dist}{=}^{\prime \prime }\mathrm{Weibul}l^{\prime \prime }). \end{array}$$

We also used logistic regression analyses to assess the relationship between survival and cognitive performance by building a GLM for binomial data with survival (survivor or non-survivor) specified as the dependent variable, the interaction between cognitive performance and sex considered as explanatory variables and the influence of body mass and age as covariates:

$$\begin{array}{rl} \mathrm{model}\;2<- & \mathrm{glm}(\mathrm{survivor}∼\mathrm{cognitive}\;\mathrm{performance}\times \mathrm{sex}\\ & +\mathrm{body}\;\mathrm{mass}+\mathrm{age},\;\mathrm{family}{=}^{\prime \prime }{\mathrm{binomial}}^{\prime \prime }). \end{array}$$

We then tested whether individual PCA composite score was correlated with survival using survival regressions with the first principal component (PC1) composite score as fixed effects, the interaction between PCA composite and sex and body mass and age as covariates:

$$\begin{array}{rl} \mathrm{model} & <-\mathrm{survreg}(\mathrm{survival}∼\mathrm{PC}1\times \mathrm{sex}\\ & \;+\;\mathrm{body}\;\mathrm{mass}+\mathrm{age},\;\mathrm{dist}={\;}^{\prime \prime }{\mathrm{Weibull}}^{\prime \prime }). \end{array}$$

## 3. Results

### (a) Individual difference in cognitive performance

#### (i) Attention task: visual attention test

Overall, 37% of 122 tested mice were attentive towards the visual stimulus at least once. Subjects varied in their total duration of attention from 0.99 to 47.16 s within the 5 min test (5.99 ± 8.19, CV = 136.93). Males had a significantly greater duration of attention compared to females (females: 4.66 ± 6.62 s; males: 7.60 ± 9.59 s; LM: *n* = 122, *F* = 3.99, *p* = 0.048; electronic supplementary material, S1, table S2 and figure S1a). There was no significant influence of age and body mass on attention (LM: *n* = 122, *F* = 0.44, *p* = 0.507; *F* = 0.16, *p* = 0.685, respectively; electronic supplementary material, table S2).

#### (ii) Problem-solving task 1: lid opening

A total of 38% of the 141 tested mice solved the task (i.e. opened at least one lid). Of these, 22% (*n* = 12) opened one lid, 26% (*n* = 14) opened two lids, 17% (*n* = 9) opened three lids and 35% (*n* = 19) opened all four lids. Males opened more lids than females (females: 0.80 ± 1.46; males: 1.19 ± 1.49; GLM: *n* = 141, *χ*^2^ = 5.55, *p* = 0.018; electronic supplementary material, S1, table S2 and figure S1b). Heavier and older mice opened significantly more lids (GLM: *n* = 141, *χ*^2^ = 14.99, *p* < 0.001; *χ*^2^ = 16.01, *p* < 0.001, respectively; electronic supplementary material, table S2).

#### (iii) Problem-solving task 2: escape box

All test mice (*n* = 135) opened the door for three consecutive trials. Some mice (*n* = 13) failed to open the door at their first attempt and hence needed between four to six trials to successfully open doors for three consecutive trials (problem-solving criterion, 3.12 ± 0.43, CV = 13.77). There was no significant influence of sex, age and body mass on success (electronic supplementary material, S1, table S2 and figure S1c).

#### (iv) Learning and reversal learning tasks

All test subjects (*n*= 87) learnt to choose the correct door for at least 10 out of 12 consecutive trials (i.e. learning criterion) within 13.48 ± 3.13 (range: 12–23) trials. Within trials, mice showed a mean success of 85% (range: 34–100%). Females tended to show a greater percentage of success compared to males (females: 86.77 ± 13.24; males: 85.13 ± 14.43; GLM: *n* = 87, *χ*^2^ = 3.61, *p* = 0.055; electronic supplementary material, S1, table S2 and figure S1d). Heavier mice tended to show a higher percentage of success (GLM: *n* = 87, *χ*^2^ = 3.75, *p* = 0.052; electronic supplementary material, table S2). There was no significant influence of age (GLM: *n* = 87, *χ*^2^ = 0.62, *p* = 0.431; electronic supplementary material, table S2).

We tested 78 of the 87 mice in the reversal learning task, excluding nine mice that were not re-trapped within 6 days after the learning task (i.e. the exclusion criterion). The reversal learning criterion was attained by all test subjects within 14.37 ± 2.59 (range: 12–24) trials. Mice had a mean percentage of success of 75% (range: 52–100%). There was no significant influence of sex, body mass and age on percentage of success during the reversal learning task (electronic supplementary material, table S2 and figure S1e).

#### (v) Inhibitory control task: detour test

The detour task was achieved by 93% of test subjects (*n* = 59 of 63 mice) within 3.60 ± 2.69 (range: 1–12) trials. There was no influence of sex and age on performance during the detour task (electronic supplementary material, S1, table S2 and figure S1f). Heavier mice were better performers (i.e. lower number of trials required to detour the device; LM: *n* = 63, *F* = 30.96, *p* = 0.048; electronic supplementary material, table S2).

### (b) Relationship between individual performance in different cognitive tasks

There was no significant correlation between different tasks (electronic supplementary material, S1, table S3). To explore whether variation in the cognitive performance could be explained by a composite score (analogous to *g*), we conducted a PCA and extracted three components with eigenvalues of greater than 1. Performances in all tasks, except the detour task, loaded positively onto the first component, which captured 20.4% of the total variance (electronic supplementary material, S1, figure S2). The variance of component 1 was mainly explained by problem-solving performance in task 1 (lid opening). A general intelligence factor *g* has been considered to occur when all tasks positively contribute to PC1 and explain greater than 30% of total task variance [10,11]. Thus, the results do not support a *g* score for the measured traits in our striped mouse population.

### (c) Relationships between cognitive performance and survival

Striped mice varied in number of days of survival before the long-distance dispersal season: 165.81 ± 55.01 days (range: 6–193 days; electronic supplementary material, S1, figure S3). There was no significant influence of sex and body mass on the number of days of survival (LM: *n* = 143, *F* = 0.16, *p* = 0.685; *F* = 0.58, *p* = 0.453, respectively).

Results from the survival regressions showed that mice with better performance in the problem-solving 2 task (escape box) and the inhibitory control task survived longer (survival regression: *n* = 135, *χ*^2^ = 4.36, *p* = 0.037, *n* = 31, *χ*^2^ = 4.55, *p* = 0.033, respectively; table 1). Mice which performed better in the problem-solving task 1 survived for a shorter time (survival regression: *n* = 141, *χ*^2^ = 4.03, *p* = 0.045; table 1). Males with better performance during the reversal learning task survived longer (survival regression: *n* = 40, *χ*^2^ = 4.01, *p* = 0.044; table 1).

**Table 1.** Relationship between cognitive performance and survival measured as the number of days before long-distance dispersal. (Results from the survival regressions consider the censored value of survivor (censoring status 0 = censored, 1 = dead observed) and the model is fitted on the Weibull's distribution that is used to model the increase of instantaneous risk of death over time (italics indicates *p* < 0.05).)

task	estimate	survival (number of days before dispersal)			
		s.e.	X2	d.f.	*p*
attention	−0.01	0.04	1.04	1	0.306
attention × sex	−0.03	0.04	0.22	1	0.637
attention × age	0.01	0.01	0.01	1	0.971
attention × body mass	−0.01	0.01	1.04	1	0.308
detour task	−0.14	0.06	4.55	1	*0.033*
detour × sex	−0.02	0.04	0.22	1	0.637
detour × age	0.01	0.04	0.01	1	0.947
detour × body mass	0.01	0.01	0.14	1	0.704
problem-solving 1: lid opening	−0.12	0.06	4.03	1	*0.045*
problem-solving × sex	0.23	0.22	1.07	1	0.299
problem-solving × age	0.03	0.07	0.41	1	0.683
problem-solving × body mass	0.02	0.01	1.52	1	0.129
problem-solving 2: escape box	−0.61	0.61	4.36	1	*0.037*
problem-solving × sex	−0.19	0.68	0.08	1	0.772
problem-solving × age	−0.10	0.18	0.29	1	0.587
problem-solving × body mass	−0.01	0.04	0.13	1	0.715
learning	0.01	0.01	0.07	1	0.783
learning × sex	−0.01	0.02	0.25	1	0.619
learning × age	0.01	0.01	2.21	1	0.137
learning × body mass	0.01	0.01	1.94	1	0.163
reversal learning	−0.01	0.01	1.13	1	0.288
reversal learning × sex	−0.01	0.27	4.01	1	*0.044*
reversal learning × age	−0.01	0.01	1.18	1	0.276
reversal learning × body mass	0.01	0.01	0.66	1	0.145

**Figure 2.**
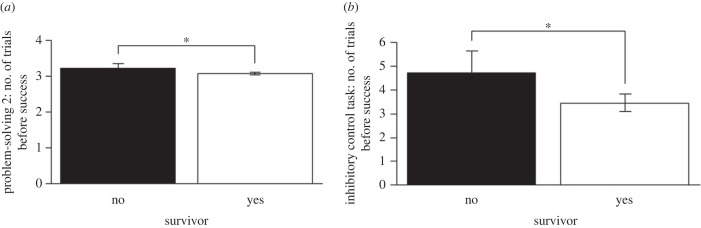
Number of trials required before success in striped mice that did (white bars) or did not (black bars) survive until the onset of the breeding season in (*a*) the problem-solving task 2 (escape box) and (*b*) the inhibitory control task (detour task). Better performance on a task was indicated by a lower number of trials. GLM, *p < 0.05. Error bars represent s.e.m.

Surviving mice showed better performance during the problem-solving task 2 (escape box) and the inhibitory control task (GLM: *n* = 135, *χ*^2^ = 2.99, *p* = 0.044, *n* = 31, *χ*^2^ = 3.88, *p* = 0.048, respectively; figure 2*a,b*). We found no significant relationship between survival and the first component score from the PCA (survival regression, *n* = 143, *χ*^2^ = 0.41, *p* = 0.520).

## 4. Discussion

We provide evidence for selection on cognitive performance in a free-living rodent population inhabiting a challenging habitat. Specific cognitive traits correlated with individual survival rate. We found that: (i) sex and body condition were associated with individual variation in cognitive performance but not survival, (ii) sex-specific differences in cognitive performance influenced survival; surviving males showed greater cognitive flexibility in a reversal learning task, and (iii) better problem-solving and inhibitory control performances were significant predictors of survival. Our study provides promising evidence for selection of specific cognitive traits and the influence of intrinsic characteristics (e.g. sex) in the evolution of cognition.

### (a) Individual differences in cognitive performance

Individual variation in cognitive performance forms the foundation for understanding the mechanisms, development and evolution of cognition [21]. Male striped mice performed better during the visual attention task and the problem-solving task 1 (lid opening), whereas females tended to show better performance during the learning task. Similarly, in a previous study, male striped mice had quicker attentional shifts towards a predator stimulus during the period with high predator risk [45]. Sex was not a significant predictor of performance during the problem-solving task 2 (escape box), the reversal learning task and the inhibitory control task. Better attention and problem-solving performances by males could be related to enhanced environmental monitoring and innovation needed by the dispersing sex (i.e. males), and are more likely to encounter novel environmental challenges [63]. A previous study showed that male striped mouse survival improved with better performance in specific cognitive traits such as spatial memory [47]. Thus, cognition in striped mice could be related to sex-specific behavioural and life-history characteristics, such as male-biased dispersal [64].

Body condition could reflect variation in energetic state and influence cognitive performance [28,65]. In the present study, striped mice with heavier body mass performed better during problem-solving task 1 (lid opening) and the learning and inhibitory control tasks. It is known that larger, more competitive individuals innovate more than smaller individuals owing to an ‘excess of energy’ availability [66]. However, this is in contrast with our expectations and those of other studies purporting the ‘necessity drives innovation’, in which young, low-ranking individuals in poorer body condition were more likely to innovate [67]. A similar explanation can also apply to the detour task, which appears to be crucial in behavioural flexibility and innovation [17,68]. Finally, since body condition is a good proxy for the energetic state of free-living individuals [28], a heavier body mass could have enabled striped mice to cope with the energetic challenges i.e. dry season with reduced food availability and performed better in energetically demanding cognitive functions such as problem-solving, learning and inhibitory control (e.g. [44]).

### (b) Relationship between individual performance in different cognitive tasks

In our study, performance in any of the cognitive tasks was a relatively poor predictor of performance in any of the other tasks. In addition, the PCA analysis showed that three factors had eigenvalues greater than 1 and not all tasks loaded positively onto PC1. PC1 explained 20.4% of the variance, less than what was reported in laboratory mice (22–38% e.g. [9,10]), birds (34–65%, e.g. [11,13]) and humans (40% e.g. [61]). A *g* factor exists when all tasks positively contribute to PC1 and explains greater than 30% of variance [10,11]. Thus, our results suggest that a *g* factor could not be detected in our study population. One explanation for the lack of evidence for a *g* factor could be related to the adaptive significance of spatial cognition in striped mice, since they have to learn and remember the spatial location of foraging sites and find shelter during daily foraging and also during dispersal [47]. The escape box and the detour tasks both measure cognitive abilities in a spatial context. Previous studies in other species suggested the existence of a spatial cluster in a test battery performance and that spatial tasks tend to correlate with just each other on a single navigation factor but often do not correlate with general intelligence [19,69,70]. Thus, a modular view of cognitive abilities may better explain the variance in the task performance of mice and hence, indicates that cognition includes independent modules that are domain-specific in striped mice (e.g. [19]). Further testing using a battery of tests that are non-spatial would help elucidate whether a lack of a *g* factor in the present study is owing to the possibility that spatial cognition is a separate construct from general intelligence.

### (c) Relationships between cognitive performance and survival

Performance in problem-solving, inhibitory control and reversal learning were significant predictors of survival of striped mice. By contrast, no general intelligence score was predictive of survival. When testing these particular cognitive traits, there is some agreement in the literature that they could reflect overall cognitive flexibility [43]. Cognitive flexibility is important for species experiencing unpredictable environments. For example, learning flexibly is useful for locating new foraging sites [34]. Our striped mice population faces unpredictable seasonal changes in food availability [39] and frequent and intensive droughts. Cognitive flexibility seems to be advantageous for individual striped mice under these conditions since problem-solving and reversal learning are quicker during harsher conditions [44,46].

Problem-solving, tested using an escape box task to reach the nest, was positively correlated with survival, whereas problem-solving tested with a lid opening task to reach food was negatively correlated with survival. This raises the question of the ecological relevance of the tasks. In striped mice, reaching the safety of cover/shelter at the nest is frequently observed in the field. Yet, the nest entrance is unlikely to have impeding obstacles. Thus, the novelty of the situation (pass through an obstacle) and behavioural necessity (reach the nest for cover) seemed pertinent for testing innovative problem-solving [67,71]. By contrast, performance in the lid opening task to reach food could be related to the influence of individual variation in food motivation and an overall lack of ecological relevance since it was not tested in the field [72]. This also raises the question of the influence of non-cognitive factors such as personality, motivation, perceptual biases and prior experience [7]. One could argue that foraging in a novel context requires some degree of risk-taking behaviour. Thus, bolder, more active individuals may try to maximize reward, at the cost of encountering higher survival risks [73]. Inhibitory control, tested with the detour task, mimicked natural situations where the animal must temporarily move away from and then find an indirect route to a goal. Several cognitive functions are thought to influence the ability to accomplish the detour behaviour, including spatial learning abilities, reasoning and inhibitory motor control [74]. We used a transparent barrier detour task to particularly study inhibitory motor control. It appears that the spatial context of the problem-solving task 2 (escape box) and the inhibitory control task (detour task) may also reflect the adaptive significance of spatial abilities in striped mice.

Striped mice demonstrate sex-specific differences in cognitive performance, depending on environmental harshness [44,46], which influences survival [47]. Female survival has been correlated with faster attention to a predator stimulus, whereas male survival has been correlated with greater spatial memory [47]. In the present study, male survival was predicted by better reversal learning performance. Other studies have not considered (or found) a sex effect in the relationship between cognition and survival [75]. Further studies are needed to understand how cognitive traits are shaped by natural and/or sexual selection.

## 5. Conclusion

To show that animal cognition evolves by natural selection requires that: (i) there is variability in cognition between individuals, (ii) that this variability in cognitive performances is heritable, and (iii) that this variation is related to variance in fitness (survival, reproductive success) under specific environmental conditions [76]. We provide evidence for variability in cognition by sex and/or body condition and that this variation is related to survival under natural conditions. Thus, there appears to be selection for specific cognitive traits, such as problem-solving, reversal learning and inhibitory control, in a rodent population facing challenging environmental conditions. Problem-solving, reversal learning in males and inhibitory control cognitive traits were better predictors of survival compared to a general intelligence score. This concurs with the idea that a set of dedicated cognitive modules may be under strong selection [3,37]. Further studies are needed to evaluate whether variation in cognitive traits is heritable to assess whether selection favours particular traits. Such studies will also help reveal how different cognitive functions arise and the constraints in the evolution of more complex cognition traits.

## Data Availability

The dataset supporting this article has been uploaded as part of the electronic supplementary material. Electronic supplementary information corresponds to additional tables and figures, cognitive tests videos and dataset [77].
